# Denis Mukwege: Advocacy Within and Beyond the Operating Room

**DOI:** 10.7759/cureus.100348

**Published:** 2025-12-29

**Authors:** Audrey Amarachi Ojinnaka, Florence Odaka, Rahul Kashyap

**Affiliations:** 1 Medical School, Touro College of Osteopathic Medicine, New York, USA; 2 Medical School, Trinity School of Medicine, Warner Robbins, USA; 3 Department of Research, WellSpan York Hospital, York, USA

**Keywords:** access to obgyn care, democratic republic of congo, general obgyn, historical vignette, nobel laureate, rape, sexual & gender based violence (gbv/sgbv), trauma injuries

## Abstract

Dr. Denis Mukwege is a Congolese gynecologist, human rights activist, and Nobel Peace laureate. He is the world’s leading specialist in reconstructive surgeries of sexual violence as a weapon of war, having treated thousands of victims. His global efforts against the use of rape as a weapon of war were recognized with the award of the Nobel Peace Prize in 2018, among other honors. Dr. Mukwege’s advocacy and reconstructive surgeries have left an indelible mark on women’s health, echoing through the corridors of human rights and reshaping the narrative of empowerment and self-care.

## Introduction and background

**Figure 1:**
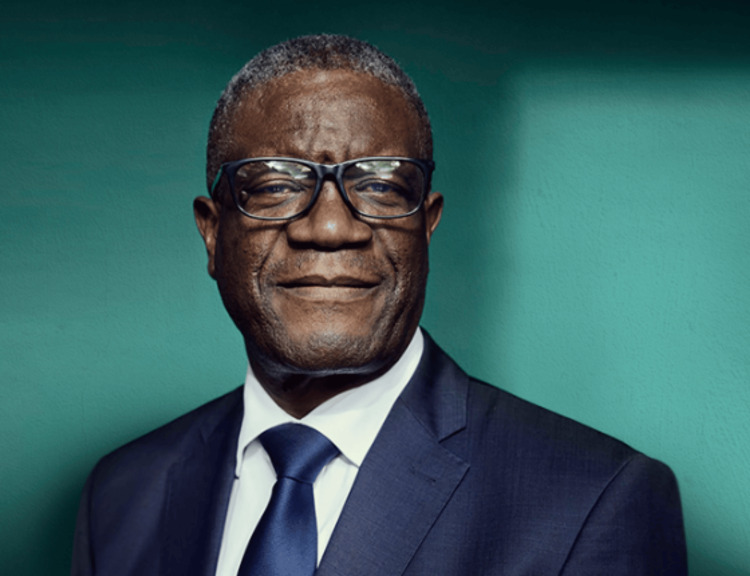
Dr. Denis Mukwege, 2024 Aurora Prize Laureate. Credit: Aurora Prize (auroraprize.com).

The purpose of this article is to highlight the ongoing legacy of Dr. Denis Mukwege, whose contributions to the specialty of gynecology and his fearless work as an advocate of women’s rights in the war-torn Democratic Republic of Congo continue to provide a catalyst for change in the region (Figure 1). The death toll as a result of the First (1996-1997) and Second (1998-2003) Congo war is estimated at more than five million, where the majority of deaths are linked to displacement, crowding, malnutrition, disease, and violence [1]. Sexual violence, specifically, rape as a weapon of war, was a prominent tactic to coerce and instill fear in invaded communities. An estimation of over 50,000 sexual violence crimes were reported across almost half the health centers in the Democratic Republic of Congo in 2006 [2]. A retrospective study found that the majority of these violent acts were committed against women (96%), while women were performing daily domestic activities (48.7%) [2]. Women were gang raped (47.5%), most often committed by unknown armed men (87.2%), and survivors of these violent war crimes were detained or kidnapped post-rape (18.5%) in the area of Itura, Congo [2]. Political unrest provided an incubator for the construction of a safe haven for women’s health by Dr. Mukwege and advocacy, both recognized on a communal and global scale. It is with the hope that continued international acknowledgment will galvanize additional effort to end the heinous acts of war crimes against women and girls that are causing generational consequences.

## Review

Mukwege’s life and career

Dr. Denis Mukwege was born on March 1, 1955, in Bukavu, Belgian Congo. In his first days of life, Mukwege nearly succumbed to septicemia [3]. It was with the help of a local Swedish mercenary, Majken Bergman, that he was able to obtain the care he needed after his mother was denied help or service for her sick infant. Complications surrounding childbirth have impacted the Mukwege family for generations, as a similar theme led to the loss of both his paternal and maternal grandmothers [3,4]. It is no shock that the catalyst that paved his way to medicine surrounded this same theme. In his early life, he frequently accompanied his father, a pastor, to visit sick community members. However, during one of those visits, after rendering advice and prayers to a woman and her ailing newborn, Mukwege felt dissatisfied with the outcome of their visit [3-8]. These harrowing experiences and his understanding of the limitations of pastoral care led to his early decision that he would be a healer of the sick.

Professional challenges and milestones

**Figure 2:**
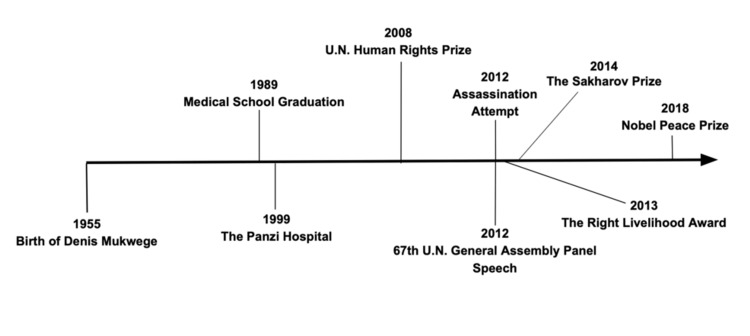
Timeline of major milestones in Denis Mukwege’s life.

Depicted below is a timeline of major milestones of Dr. Denis Mukwege's life highlighted in this article (Figure 2).

Lemera Hospital (1989-1998)

After graduating with a degree in medicine from the University of Burundi, Dr. Denis Mukwege pursued a specialization at the University of Angers in France. He later returned home to the Democratic Republic of Congo to assist in the efforts of healing. In 1989, after Dr. Mukwege graduated from medical school, he decided to return to South Kivu to work as a pediatrician at Lemera Hospital, which was about 100 kilometers from his hometown, Bukavu. Dr. Mukwege became the hospital’s medical director in 1992 and continued to work as the medical director until the outbreak of the First Congo War in 1996 [3-7]. However, with the outbreak of the First Congo War in Zaire in 1996, Dr. Mukwege found himself shifting his focus of care. During this war, Lemera Hospital became the prominent medical center providing medical care to wounded soldiers, refugees, and local civilians [9]. On October 6, 1996, Lemera Hospital was attacked by members of an armed group known as the Banyamulenge. This armed group of people stole many medical supplies and killed numerous patients whom Mukwege cared for [3,9,10]. The tragedy profoundly affected Dr. Mukwege, and he spent years working to recover from its impact. In the wake of this event, Dr. Mukwege decided that he could no longer continue to practice at Lemera Hospital and sought safety in Nairobi, Kenya, in 1997 [3-7,11,12]. Though shaken by the violence, he refused to give up on his mission to help people in eastern Congo. In 1998, he returned to his hometown, Bukavu, and started building Panzi Hospital, a small clinic focused on childbirth and emergency surgeries. Tragedy again occurred when the hospital was attacked, looted, and badly damaged by armed groups. Despite significant adversity and limited resources, Dr. Mukwege initiated the reconstruction of the Panzi Hospital [12].

The Foundation of the Panzi Hospital and the Years Following (1999-2009)

**Figure 3:**
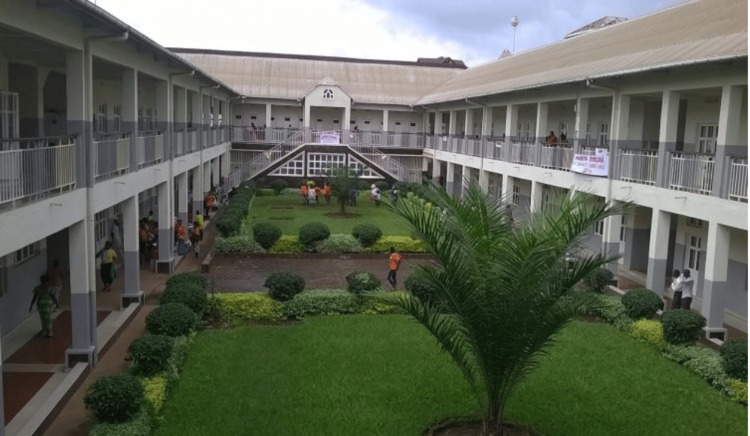
Panzi Hospital, Bukavu, Democratic Republic of the Congo. Credit: Permission obtained from Make Music Matter.

By 1999, with Panzi Hospital rebuilt and operating, Dr. Mukwege recognized a growing crisis: many women lacked a safe place to give birth. This urgent need inspired him to formally establish Panzi Hospital that year, with crucial support from the Community of Pentecostal Churches in Central Africa (CEPAC) [3-7,9,13]. Initially, the hospital was commissioned with just two rooms, hoping to conduct deliveries and cesarean sections for women to reduce maternal mortality. However, the first woman who was treated at the hospital was not a delivering mother but a rape victim who had been shot in her genitals [3,5,7,14]. At first, Dr. Mukwege thought this act was committed by some barbaric individual, but as many more similar cases came in during that period, he and his team soon realized that these women and girls were survivors of a war that was still ongoing in the region. The number of rape survivors who came to seek care at the Panzi Hospital increased daily in the wake of devastation in the community, thereby transitioning the hospital that was meant to be a maternity ward to a center for treating war-related gynecological trauma. The hospital grew to have a capacity of 125 beds during that time [11]. Currently, the hospital has a capacity of 450 beds, also specializing in reconstructive surgery of the female genitalia and gynecological pathologies like lower urogenital and digestive fistulas (Figure 3) [3,7,9,15-17].

In addition to the establishment and progression of the Panzi Hospital during this time, Dr. Mukwege has been recognized by his professional community several times throughout his career. Namely, he was awarded the United Nations Human Rights Prize in 2008 for his outstanding contribution to the promotion and protection of human rights and fundamental freedoms.

Community impact

Dr. Mukwege’s Research, Advocacy, and Resilience (2010-2019)

Physical and psychological trauma were recurring themes in this war-riddled region during this time. A study, in which Dr. Mukwege assisted, outlined that 255 women who reported to Panzi Hospital and two neighboring community-based clinics experienced some level of sexual and gender-based violence. The report found that 76% women/girls reported being raped, 69% described being gang raped, and 83% indicated that the assailant(s) were wearing military uniforms [18]. The article also narrated that rejection from the community (7%), as well as even one’s own family (29%), was a potential outcome for sexual and gender-based violence victims. A retrospective study conducted by Dr. Mukwege on the patients of the Panzi Hospital highlights that of 1,021 medical records, 59% of attacks happened at night and 57% happened in the victim’s home [19]. Victims of these assailants experienced aftermaths riddled with a multitude of adverse reactions, including, but not limited to, infertility, social isolation, vulnerability, and in some cases, the end of relationships [20].

Dr. Mukwege's research exposed the psychosocial and public health consequences of sexual and gender-based violence in eastern Congo, which provided a platform for expansion to international advocacy efforts. Dr. Mukwege attended the 67th United Nations General Assembly panel discussion, “Preventing Sexual and Gender-Based Crimes in Conflict and Securing Justice for Survivors” on September 25, 2012. He brought to this global stage the atrocities that were occurring in his country, and he courageously highlighted the urgent need for the Democratic Republic of Congo’s government to address these crimes. One month later, five armed men held his family at gunpoint in an attempt to assassinate him in his home. His beloved friend and guard lost his life while protecting Dr. Mukwege and the Mukwege family as they escaped the country for safety.

**Figure 4:**
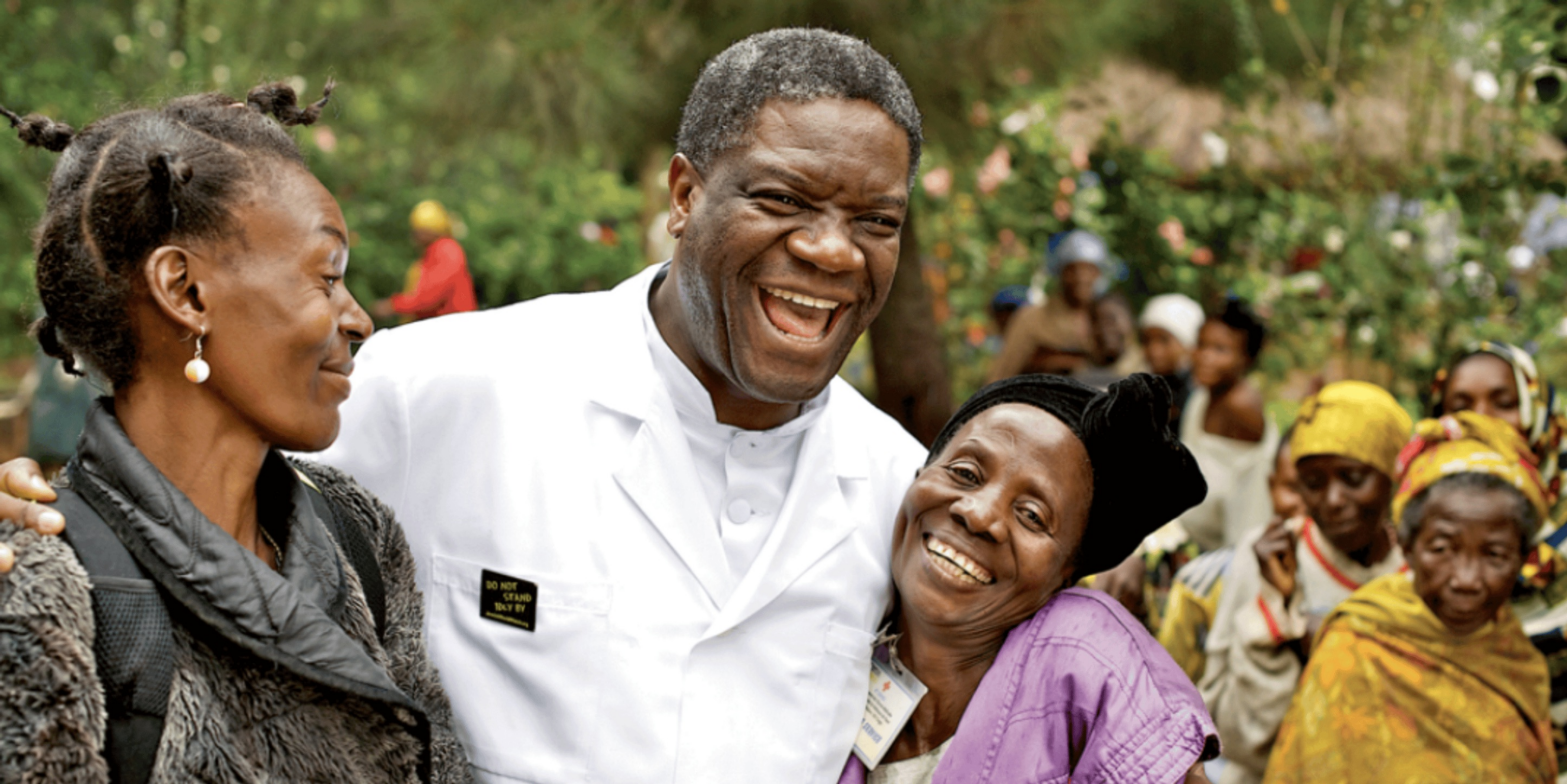
Dr. Denis Mukwege with women from the local community. “Dr. Denis Mukwege: the man who repairs women victims of sexual violence.” Credit: Permission obtained from the Panzi Foundation.

After being forced to flee from the Democratic Republic of Congo in 2012, his absence in the community and at the Panzi Hospital was felt by many. In his absence, the women in the local community who were repeatedly treated by Dr. Mukwege organized around-the-clock security and funds so that he could return and continue his work. The heartfelt effort of the women led to his return to the Panzi Hospital one year later, amidst continuous threats on his life, wartime angst, and all attempts to silence his advocacy efforts on a worldwide scale (Figure 4).

Dr. Mukwege received numerous awards for his commitment to women's rights, including the Right Livelihood Award (2013) for his courageous work healing women survivors of wartime violence and speaking up about the root cause, the Sakharov Prize of the European Parliament (2014) for freedom of thought and his fight for the protection of women, and finally, he co-received the Nobel Peace Prize (2018) alongside Nadia Murad for their efforts to end the use of sexual violence as a weapon of war and armed conflict, among others.

## Conclusions

Dr. Denis Mukwege’s legacy is one that is marked by fearlessness and the relentless commitment to the cessation of the maltreatment of women internationally. He has directly affected the lives of women in the tens of thousands with his pioneering reconstructive surgeries for genital mutilation. In addition, his advocacy for international law and policy reform on behalf of women at global forums, including the United Nations, as well as his recognition with the Nobel Peace Prize, has drawn international attention to the consequences faced by women and their families in the Democratic Republic of the Congo and other conflict-affected nations. Unfortunately, sexual violence as an act of war has yet to be eradicated. Through continued transnational acknowledgement, we can further strive to restore dignity and respect to women globally.
